# Different evolutionary histories of two cation/proton exchanger gene families in plants

**DOI:** 10.1186/1471-2229-13-97

**Published:** 2013-07-04

**Authors:** Inês S Pires, Sónia Negrão, Melissa M Pentony, Isabel A Abreu, Margarida M Oliveira, Michael D Purugganan

**Affiliations:** 1 Instituto de Tecnologia Química e Biológica, Universidade Nova de Lisboa, Av. da República, 2780-157 Oeiras, Portugal and iBET, Apartado 12 2781-901, Oeiras, Portugal; 2 Department of Biology and Center for Genomics and Systems Biology, New York University, New York, US

## Abstract

**Background:**

Gene duplication events have been proposed to be involved in the adaptation of plants to stress conditions; precisely how is unclear. To address this question, we studied the evolution of two families of antiporters. Cation/proton exchangers are important for normal cell function and in plants, Na^+^,K^+^/H^+^ antiporters have also been implicated in salt tolerance. Two well-known plant cation/proton antiporters are NHX1 and SOS1, which perform Na^+^ and K^+^ compartmentalization into the vacuole and Na^+^ efflux from the cell, respectively. However, our knowledge about the evolution of *NHX* and *SOS1* stress responsive gene families is still limited.

**Results:**

In this study we performed a comprehensive molecular evolutionary analysis of the NHX and SOS1 families. Using available sequences from a total of 33 plant species, we estimated gene family phylogenies and gene duplication histories, as well as examined heterogeneous selection pressure on amino acid sites. Our results show that, while the *NHX* family expanded and specialized, the *SOS1* family remained a low copy gene family that appears to have undergone neofunctionalization during its evolutionary history. Additionally, we found that both families are under purifying selection although *SOS1* is less constrained.

**Conclusions:**

We propose that the different evolution histories are related with the proteins’ function and localization, and that the NHX and SOS1 families are examples of two different evolutionary paths through which duplication events may result in adaptive evolution of stress tolerance.

## Background

It has been argued that gene duplications underlie mechanisms to achieve stress adaptation [1,2]. There is little evidence, however, to support this. Additionally, it is not known if there is one or multiple ways of achieving stress adaptation through gene duplication. To address this question, we focused our attention on salt tolerance, and the evolutionary histories of two gene families involved in this trait.

Salt tolerance is a complex trait that is thought to have originated multiple times in plants [3], and it is important to understand the nature and molecular evolution of salt tolerance mechanisms throughout the history of land plants. This will be key not only to our understanding of how plants adapt against the disruptive effects of high salt concentrations in the soil, but also to point out directions for possible crop improvement. Indeed farmers are increasingly facing loss of crop production due to this abiotic stress, since currently > 20% of the world’s arable land is affected by high salinity [4].

Cation/proton exchangers are essential to the normal function of the cell. Besides helping regulating internal pH, cell volume, and cytoplasmic ion homeostasis [5-7], these transporters have also been shown to be involved in vesicular trafficking and protein targeting [8,9]. In plants, Na^+^,K^+^/H^+^ antiporters are also associated with salt tolerance [10,11], and a series of studies have targeted these cation exchangers to improve agronomically important crops [12,13].

All Na^+^,K^+^/H^+^ exchangers belong to a superfamily of monovalent cation/proton antiporters (CPA) and are divided in two families, CPA1 and CPA2 [14]. The CPA1 family is composed of two main groups, one containing plasma membrane-bound proteins and the other intracellular proteins [14]. Within these two groups are well-characterized antiporters associated with salt tolerance in plants, the salt overly sensitive 1 (SOS1) and sodium hydrogen exchanger (NHX) proteins. SOS1 proteins are localized in the plasma membrane [15,16], and are responsible for Na^+^ efflux from the cell. NHX proteins, on the other hand, are intracellular proteins that compartmentalize Na^+^ and K^+^ in the vacuole [17-19].

In *Arabidopsis thaliana*, the NHX protein family has six members that are classified into two classes [17]. The class I proteins, AtNHX1-4, are localized in the tonoplast [17] and have equal affinity for Na^+^ and K^+^[7,20]. Class II proteins include AtNHX5 and 6, which are localized in the endosomal compartment of the Golgi and trans-Golgi network [18] and have a higher affinity for K^+^ compared to Na^+^[6].

Previous reports have shown that the Arabidopsis *NHX* genes which encode these proteins have different expression patterns and responsiveness to abiotic stresses [21,22]. *AtNHX1* and *2* were shown to be expressed at high levels in all plant tissues, while *AtNHX3* and *4* were mainly expressed in root and flower tissues, respectively [21,22]. *AtNHX5* was also expressed in all tissues but at lower levels [21,22], while *AtNHX6* expression was detected only in shoots and roots by RT-PCR [22]. In addition, while *AtNHX1*-3 were shown to be responsive to both salt stress and abscisic acid (ABA) [22,23], *AtNHX5* was only responsive to salt stress, suggesting that its response is ABA-independent [22]. Moreover, all *NHX* genes promoted the recovery of a salt sensitive yeast mutant [21,22] and numerous studies have shown that overexpression of Arabidopsis *NHX1* and *5*, or rice *NHX1* (among others), resulted in increased salt tolerance in transgenic plants [10,24-27].

The other protein that characterizes this cation/proton exchanger family is SOS1, which belongs to the well-known salt tolerance Salt-Overly-Sensitive (SOS) pathway [28-30]. Salt stress elicits a cytosolic calcium signal, which functions as a major secondary-messenger signalling molecule. A myristoylated calcineurin B-like protein (SOS3) senses the salt-elicited calcium signal, and upon Ca^2+^ binding SOS3 undergoes dimerization and enhances the serine/threonine protein kinase activity of SOS2. The SOS3/SOS2 complex is targeted to the plasma membrane and activates SOS1 [28-30].

SOS1 has been called NHX7 by several authors, and was thus thought to be part of the NHX gene family [12,16]. Previous studies, however, regarding the phylogeny of cation/hydrogen transporters [14,31] suggest that it is distinct from the other NHX proteins and should more appropriately retain its designation as SOS1. For this reason, we refer to the Arabidopsis proteins SOS1/NHX7 and NHX8 (highly similar to, but shorter than SOS1) as SOS1A and SOS1B, respectively. SOS1B, like SOS1A, is localized to the plasma membrane [15,16]. While SOS1A is a Na^+^/H^+^ antiporter [32], however, SOS1B seems to only transport Li^+^[16]. In addition, the tonoplast exchangers in Arabidopsis are also regulated by the SOS pathway, specifically by SOS2 [33], thus suggesting coordination between tonoplast and plasma membrane antiporters.

The importance of these two families of proteins in plant salt tolerance is well established. By promoting the efflux of Na^+^, SOS1A helps protect the cell from the deleterious effects of this ion. Additionally, SOS1A seems to have an important role in long-distance Na^+^ transport, thus helping to regulate the Na^+^/K^+^ ratio in roots and shoots [17]. On the other hand, NHX family members have been described as essential to Na^+^ compartmentalization in the vacuole, protecting the cell from the deleterious effects of this ion and maintaining cytoplasmic ion homeostasis [10,34]. Recent studies, however, suggest instead that increased salt tolerance of *NHX* over-expressing plants result from an improved ability to retain K^+^ after stress induction [35-37].

Despite the key roles these genes play in salt tolerance in plants, little is known about the evolutionary histories of both gene families. Understanding the evolution of Na^+^,K^+^/H^+^ antiporters can help clarify the mechanisms leading to plant stress adaptation associated with gene duplication events [1,2,38]. In this study, we report a comprehensive molecular evolutionary analysis of both the NHX and SOS1 plant protein families. We reconstructed the phylogeny and the history of duplication events for each family, as well as determined the selection pressure on amino acid sites within these proteins. Our purpose here is not to identify novel *NHX* and *SOS1* plant genes, or to do a phylogeny analysis of the entire monovalent CPA gene family, as in others [39]. Instead, we show that the *NHX* and *SOS1* gene families have very different evolution trajectories and suggest that these divergent evolutionary histories are related to the evolution of their function and cellular localization. Finally, we suggest that the NHX and SOS1 families represent examples of two different paths in the molecular evolution of stress tolerance.

## Results and discussion

### Phylogeny of the NHX family exhibits multiple independent duplication events

**Figure 1:**
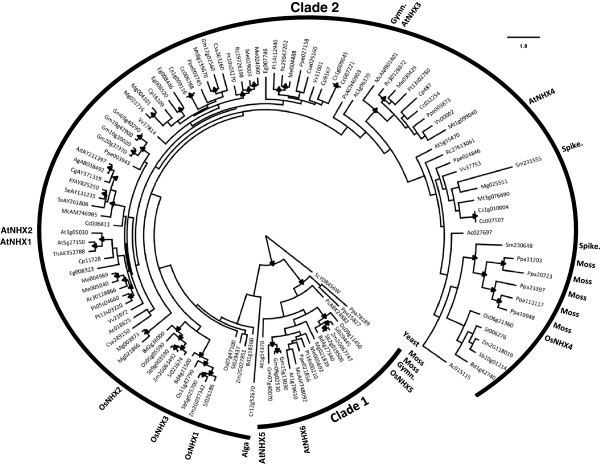
**Maximum likelihood phylogeny of the NHX family. **Nodes marked with black filled triangles represent nodes with bootstrap support > 75% (see Additional file 1: Figure S1 for more details). The root was placed using the yeast NHX protein as outgroup. The two main clades and Arabidopsis, rice, gymnosperm (Gymn.), spikemoss (Spike.), moss, alga, and yeast sequences are highlighted. (See Additional file 2: Table S1 for sequences’ codes).

We selected 121 genes in 33 taxa that appear, or are described, to belong to the NHX family. The distribution of *NHX* genes among the various species is shown in Table 1. Among these genes, 108 are found in 28 angiosperm species, 2 in 1 gymnosperm taxa, and 9 in 2 non-seed plant taxa. We also identified 1 sequence from the alga *C*. *reinhardtii*, and we used 1 sequence from the budding yeast *S*. *cerevisiae* as an outgroup. The NHX phylogeny agrees with the classification of these proteins in two distinct evolutionary groups. We obtained two main clades, with ~99% bootstrap support, which showed evolutionary divergence of NHX protein groups according to their protein localization, as previously reported [22,40] (Figure 1 and Additional file 1: Figure S1 for more detail and unrooted tree). Proteins localized in the endosomal compartment of the Golgi and trans-Golgi network grouped in clade 1 (closer to the yeast NHX1), while proteins localized in the tonoplast grouped in clade 2.

**Table 1:** Species and number of NHX and SOS1 sequences used in this study

	**Species**	**Number of putative NHX sequences**	**Number of putative SOS1 sequences**
**Yeast**	*Sacharomyces cerevisiae*	1	-
**Alga**	*Chlamydomonas reinhardtii*	1	-
**Moss**	*Physcomitrella patens*	7	2
**Spikemoss**	*Selaginella moellendorffii*	2	2
**Gymnosperm**	*Picea sitchensis*	2	-
**Angiosperms Monocots**	*Brachypodium distachyon*	5	2
	*Oryza sativa*	5	1
	*Setaria italica*	5	1
	*Zea mays*	5	1
	*Sorghum bicolor*	4	1
**Angiosperms Dicots**	*Aquilegia coerulea*	3	2
	*Mesembryanthemum crystallinum*	3	1
	*Atriplex gmelinii*	1	-
	*Atriplex dimorphostegia*	1	-
	*Suaeda salsa*	1	-
	*Salicornia europaea*	1	-
	*Chenopodium glaucum*	1	-
	*Kalidium foliatum*	1	-
	*Mimulus guttatus*	6	1
	*Vitis vinifera*	5	1
	*Eucalyptus grandis*	4	2
	*Citrus clementina*	5	1
	*Citrus sinensis*	3	1
	*Carica papaya*	4	1
	*Thellungiella halophila*	1	2
	*Arabidopsis thaliana*	6	2
	*Prunus persica*	6	1
	*Cucumis sativus*	3	1
	*Glycine max*	8	1
	*Medicago truncatula*	3	-
	*Populus trichocarpa*	6	2
	*Ricinus communis*	5	1
	*Manihot esculenta*	7	2
**TOTAL**	**33**	**121**	**32**

**Figure 2:**
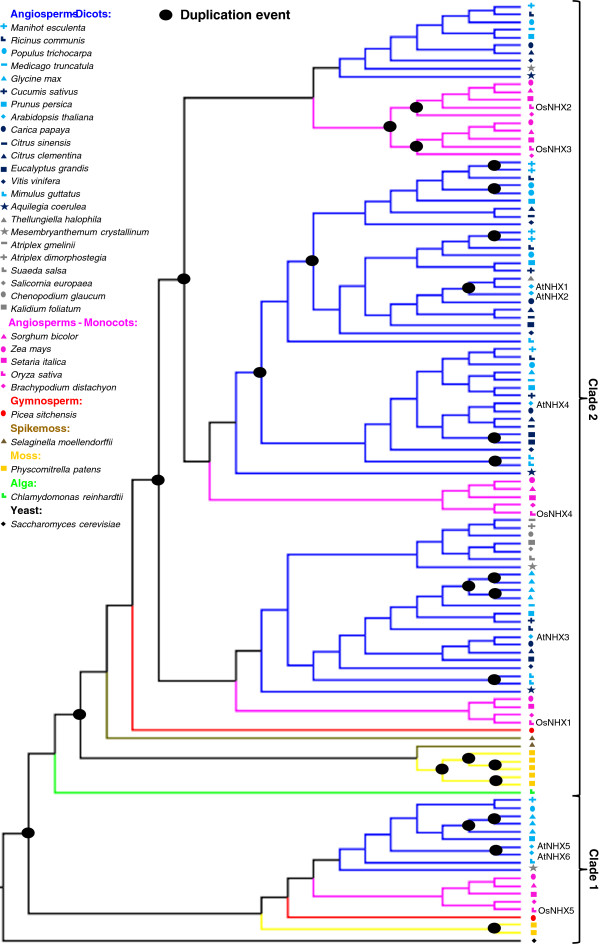
**Phylogeny of the NHX family that indicates multiple independent duplication events throughout its evolutionary history. **The reconciled NHX tree obtained using NOTUNG v2.6, has a duplication/loss score of 60.5 and shows 27 independent gene duplication and 40 gene loss (not shown) events.

We observe that multiple independent duplication events have occurred throughout the evolutionary history of the NHX family. Based on the reconciled phylogeny (Figure 2), we estimate 27 independent gene duplication and 40 gene loss events during the diversification of this gene family. Gene loss events, however, are not displayed on the phylogeny since they might not represent true losses but may be due to partial gene inventories due to incomplete whole genome sequencing data (e.g., *Thellungiella halophila*) and to some genes still remaining to be identified. In this reconciled tree, all the branches with bootstrap support inferior to a set value (here 75%) are rearranged to achieve the most parsimonious duplication and loss history for the gene family. Nevertheless our results, together with the fact that non-plant species also have multiple *NHX* genes [9,39], suggest that multiple copies of NHX proteins were already present in a common ancestor of modern land plants (even though AtNHX1-4 and their orthologs are plant specific) [39].

Interestingly, orthologs of Arabidopsis NHX1 and 2 might be specific to (at least) seed plants. NHX1 and 2 proteins consistently grouped separately from proteins from basal plant lineages such as spikemoss (*Selaginella moellendorffii*), moss (*Physcomitrella patens*), and the green photosynthetic alga (*Chlamydomonas reinhardtii*) (Figure 1 and Additional file 1: Figure S1). Due to the limited number of gymnosperm sequences (only two NHX proteins from *Picea sitchensis*) we could not assess divergence from the more closely related species to the angiosperms. Sequences from more gymnosperm species are available as EST sequences in PlantGDB database, which coupled with greater availability of genomics resource data from other non-flowering plant taxa species, could potentially be used in the future to examine more closely the precise origins of specific gene family clades. However, our results suggest that NHX proteins grouping with AtNHX1 and 2 might be more recent and specific to seed plants. Nevertheless, bootstrap supports obtained for these branches were not very high (Figure 1 and Additional file 1: Figure S1) and more sequences will be necessary to be analyzed to confirm this.

### Purifying selection on NHX proteins

We examined the sequences of the plant *NHX* gene family to determine whether there was significant heterogeneity in selective pressure among amino acid sites. Selective pressure at the protein level can be measured as dN/dS, where dN and dS are the number of non or synonymous substitutions per non or synonymous site, respectively [41]. If non-synonymous mutations are favored by positive selection, non-synonymous mutations are fixed at a faster rate and dN/dS > 1 [41]. In the case of purifying selection dN/dS < 1. However, if selection has no effect on fitness due to neutral evolution, both mutations are fixed at the same rate and dN/dS = 1 [41].

For *NHX* coding region sequences from both clade 1 and 2, the average dN/dS was estimated at ~0.08 and no sites were predicted to be under positive selection. Additionally, no difference in dN/dS between *NHX* genes was identified. We believe that these results might reflect the key function of NHX proteins, which besides salt tolerance [10,11] include normal cell functions in vesicular trafficking and protein targeting [8,9]. Additionally, NHX proteins are predicted to have ~12 transmembrane domains [42] and are probably constrained at the structural level. Our results are consistent with the purifying selection expected for these proteins [43] and agree with results recently obtained by Hudson *et al*. [44].

### Phylogeny of the SOS1 family exhibits few and recent duplication events

Unlike the NHX family, plant species appear to have lower numbers of *SOS1* genes in their genomes. We have selected 32 genes in 22 taxa that appear, or are described, to belong to the SOS1 family. The distribution of *SOS1* genes among the various species is shown in Table 1. Among these genes, 22 are found in 16 angiosperm species, and 4 in 2 non-seed plant taxa. In this case we used 2 sequences from the moss *P*. *patens* as outgroup sequences.

We found that eight plant species, including *A*. *thaliana*, seem to have two SOS1-like genes (Table 2). At the time of submission of this manuscript, however, a new BLASTp search using Phytozome v8.0 (http://www.phytozome.net/, verified in June 2012) suggested that more species might possess more than one Putative *SOS1* protein. The existence of two *SOS1* genes has been described previously for *Arabidopsis thaliana*[16] and two other plant species not included in this study, namely Neptune grass (*Cymodocea nodosa*) [45] and quinoa (*Chenopodium quinoa*) [46]. Nevertheless, to confirm that these seven species have two SOS1-like proteins like in Arabidopsis, it is necessary to perform further functional studies.

**Table 2:** **Plant species predicted to have two SOS1**-**like genes**

**Species**	**Putative SOS1 – longer gene**		**Putative SOS1 – shorter gene**		**∆ Length (AA)**
	**Sequence code**	**Length (AA)**	**Sequence code**	**Length (AA)**	
*Physcomitrella patens*	Pp1s12_8V6.1	1153	Pp1s15_101V6.1	1060	93
*Selaginella moellendorffii*	74518	1024	75049	940	84
*Brachypodium distachyon*	Bradi4g00290.1	1138	Bradi4g17380.1	719	419
*Aquilegia coerulea*	Aca_000299m	1150	Aca_002550m	588	562
*Eucalyptus grandis*	Egrandis_v1_0.001457m	1001	Egrandis_v1_0.008144m	547	454
*Arabidopsis thaliana*	At2g01980	1146	At1g14660	733	413
*Populus trichocarpa*	POPTR_0010s11130.1	1147	POPTR_0008s14030.1	1145	2
*Manihot esculenta*	cassava4.1_025141m	810	cassava4.1_002710m	703	107

∆ represents the difference in protein length.

As in Arabidopsis, the species with two SOS1-like proteins usually displayed differences in protein length (Table 2). However, two classes of length differences were identified - one in which both duplicates had similar lengths (∆ < 110 amino acid residues), and another in which the difference in duplicates length was substantially larger (∆ > 400 amino acid residues). Nevertheless, as in Arabidopsis, the differences in length were mainly due to shortening in the C-terminus instead of deletions within the protein primary sequence. This is important to notice, because it was shown by Quintero *et al*. [47] that it is within the C-terminus of SOS1A that lays the activation site and the auto-inhibitory domain of the protein. Additionally, SOS1-like proteins from *A*. *coerulea*, *E*. *grandis*, and *M*. *esculenta* have deletions in the N-terminus of the protein when compared to the Arabidopsis SOS1 proteins. This suggests that SOS1-like proteins from these three species might have a smaller transmembrane region, but further biochemical analyses need to be performed to confirm this.

**Figure 3:**
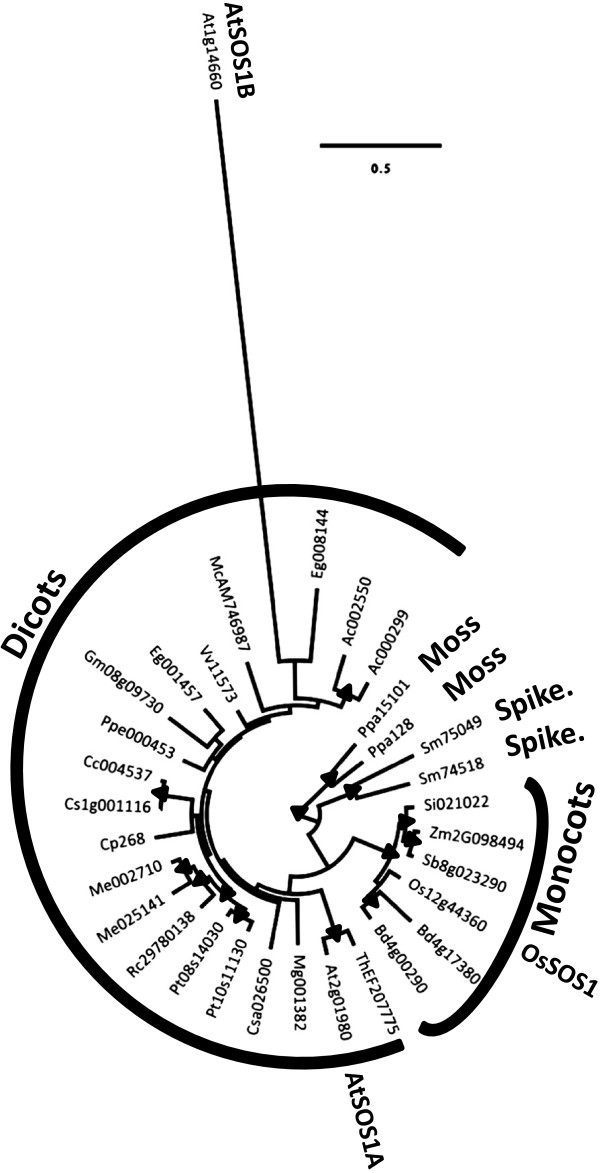
**Maximum likelihood phylogeny of the SOS1 family. **Nodes marked with black filled triangles represent nodes with bootstrap support > 75% (see Additional file 1: Figure S2 for more details). The root was placed using the Physcomitrella moss SOS1 as outgroup. The monocots and dicots clades, as well as the Arabidopsis, rice, spikemoss (Spike.), and moss sequences are highlighted. (See Additional file 2: Table S1 for sequences’ codes).

The SOS1 phylogeny (Figure 3 and Additional file 1: Figure S2) exhibits three distinct clades: (1) one that contains the moss and spikemoss proteins (~94% bootstrap support), (2) another that has monocot proteins (~91% bootstrap support), and (3) finally, one clade with dicots SOS1 sequences. The SOS1 protein phylogeny is almost identical to the accepted species phylogeny of the study species, although there appears to be some long-branch attraction that alters the position within the dicot clade of one *Eucalyptus grandis* and one *A*. *thaliana* protein.

**Figure 4:**
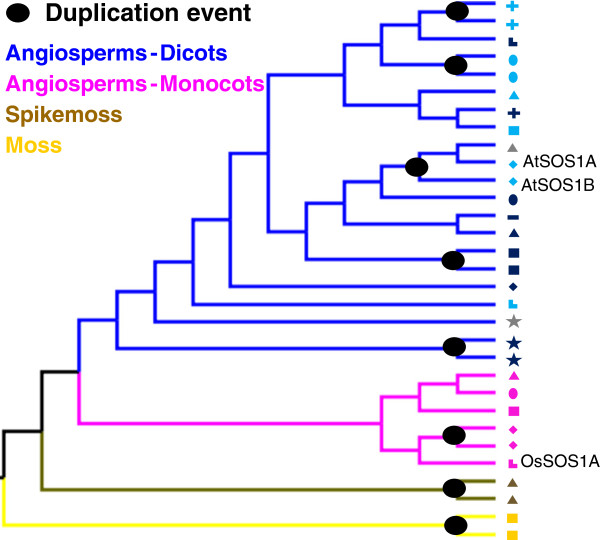
**Phylogeny of the SOS1 genes indicating few recent duplication events. **The reconciled SOS1 tree obtained using NOTUNG v2.6, has a duplication/loss score of 13.0 and shows 8 independent gene duplication and 1 gene loss (not shown) events. All duplication events appear towards the terminal nodes of the tree indicating that these events occurred recently.

Unlike the NHX family, the SOS1 family has undergone fewer gene duplication events. Reconciliation of the SOS1 tree with the species tree (Figure 3 and Additional file 1: Figure S3) estimates that 8 independent gene duplication events and one loss have occurred within the land plants (Figure 4). Once again, gene losses may simply represent unidentified genes; in fact Oh et al. [2] recently reported that *Thellungiella parvula* has three copies of the *A*. *thaliana* SOS1B and that these result from recent tandem duplications.

Interestingly, gene duplication events in the SOS1 family appear toward the terminal nodes of the reconciled tree, indicating that these events were recent in evolutionary time. The analyses of some species that have a second copy of SOS1 further support this observation. For example, *Populus trichocarpa* is thought to have undergone a recent whole genome duplication event [48] and monocots such as *Brachypodium distachyon* are thought to be more prone to tandem duplications which suggests that any gene duplication in one monocot species is probably recent and not shared with the common ancestor of this group [49].

### Purifying selection on SOS1 proteins with few amino acids under positive selection

**Figure 5:**
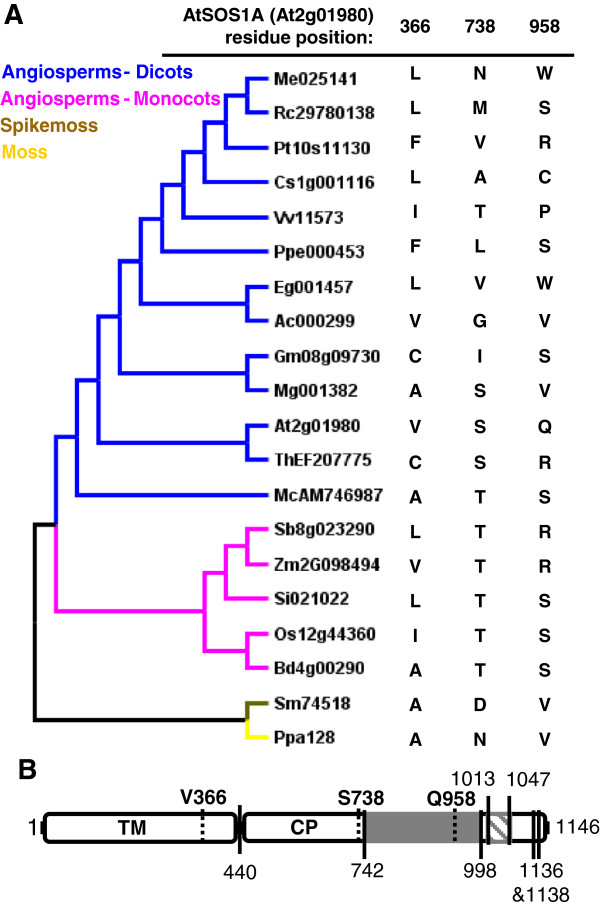
**Amino acid sites under neutral or positive selection in SOS1A. **SOS1 proteins are generally under purifying selection but appear to have some residues under neutral or positive selection. However, these residues do not fall in the auto-inhibitory domain or in sites important for the activation of the protein. **A **– Amino acids under neutral or positive selection for each SOS1A ortholog, which are shown in the NJ tree on the left. **B **– *A*. *thaliana *SOS1A protein diagram based on Figure 2A and information obtained by others [47]. Amino acid residues predicted to be under neutral or positive selection are indicated by the broken lines. The grey area (residues 742–998) corresponds to the target-region of the auto-inhibitory domain, which is highlighted in dashed grey (residues 1013–1047). Amino acid residues 1136 and 1138 correspond to sites of recognition and phosphorylation, respectively, by the SOS2-SOS3 complex. TM: Trans-membrane region (residues 1–440). CP: Cytoplasmic region (residues 441–1146).

Like the NHX proteins, the SOS1 proteins are generally subject to purifying selection, but our analysis indicates that some amino acid sites appear to be under positive selection. Among the land plants the SOS1 proteins had an average dN/dS of ~0.16. Using alignments that largely represented orthologous sequences, the glutamine at position 958 (Q958) in *A*. *thaliana* is evolving neutrally (dN/dS ~ 1.00) and two amino acid residues are under positive selection (dN/dS ~ 1.23) - a valine at position 366 (V366) and a serine at position 738 (S738) (Figure 5). Other residues (L843, C902, and P915 in *A*. *thaliana*) were identified in our analysis as strong candidate residues to be under neutral or positive selection, but these were not as well supported as the other three sites. Nevertheless, these other residues might be indeed under neutral or positive selection since the tests designed to detect positive selection are very stringent.

In general, structurally constrained sites should be under stronger purifying selection, while unstructured sites have higher levels of amino acid replacements [43]. According to different prediction tools available at ExPASy (http://expasy.org/tools/), however, the positively selected residue V366 is in a predicted transmembrane helix. Amino acid site Q958 is predicted to be in a transition zone between coiled and beta-sheet regions, and residue S738 is predicted to be in a random coil.

Two of these residues, V366 and S738, are in the cytoplasmic domain of AtSOS1A (Figure 5) but do not fall in the auto-inhibitory domain (amino acids 1013–1047) or near sites known to be relevant for protein activation by phosphorylation (S1138 and S1136) [47]. Additionally, mutations of these three residues under neutral or positive selection are not predicted to result in changes that affect secondary or tertiary structure, or create new sites for putative posttranslational modifications (data not shown). Nevertheless, residue S738 is in close proximity to residues S742 and V743, which were shown to affect the activity of the *A*. *thaliana* protein when mutated [47]. Like S742 and V743, S738 is not well conserved between species. Mutations within the transmembrane pore were also shown to affect AtSOS1A activity [47]. Thus, it may be that the roles of residues S738 and V366 in the activity of the SOS1A protein are worth study.

### Relaxation of selection on SOS1 gene duplicates

The SOS1 family apparently exhibits a relaxation of purifying selective pressure upon duplication, as previously reported in other gene families [50] and, specifically for ion transporters [44]. This relaxation of selection, however, seems to be present in only one duplicated gene copy, and only in species that have two SOS1-like proteins differing in length. Although the free-ratio model [51] is not generally considered to be a good method to predict dN/dS because it has too many free parameters, the comparison between values of dN/dS is suggestive (Additional file 1: Figure S4). Species which have two Putative *SOS1*-like proteins similar in length (∆ < 110 amino acid residues) showed a difference in dN/dS values less than 0.1, while species that retain two putative *SOS1*-like proteins with very different length (∆ > 400 amino acid residues) displayed a difference in dN/dS values always greater than 0.2. Furthermore, in the cases where the two putative *SOS1*-like proteins substantially differed in length, the shortest appeared to be evolving faster than the longer protein from the same species (Additional file 1: Figure S4). We also confirmed that these results were not affected by a saturation of nucleotide substitutions on the shorter sequences (Additional file 1: Figure S5), except possibly for At*SOS1B*.

More data is needed to verify if this trend is statistically significant. Nevertheless, we can speculate that the possible relation between the differences in protein length and dN/dS might be due to the relative recent occurrence of the duplications in *SOS1*, which could indicate that not enough time has elapsed for differentiation between duplicated genes to have occurred (e.g., woody plants, such as Populus, have a slower evolution rate than Arabidopsis) [48]. Another hypothesis to explain the different dN/dS values observed between *SOS1* proteins that differ in length is that the duplication of some *SOS1* genes did not result in functionally equivalent gene copies, and hence functional divergence between copies is immediately observed.

## Conclusions

### Contrasting evolutionary histories between the *NHX* and *SOS1* gene families

**Figure 6:**
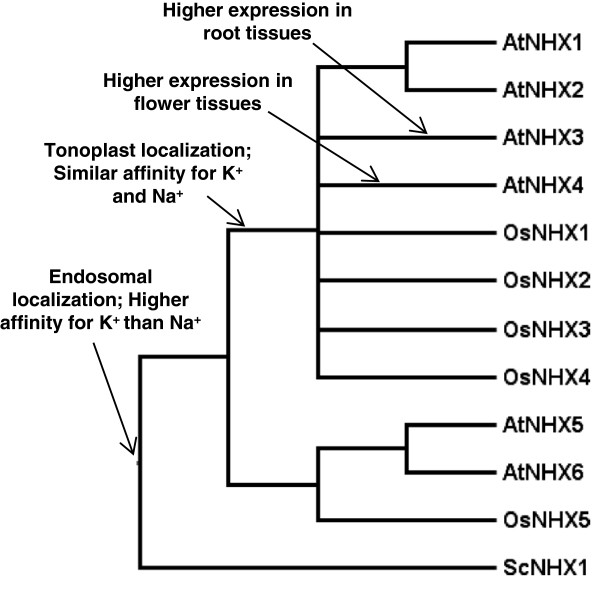
**Protein subcellular relocalization and spatial subfunctionalization events occurred during the evolution of the NHX family. **NHX family has undergone a series of protein subcellular relocalization and spatial subfunctionalization events. These events are clearly observed by phylogenetically mapping functional and expression information collected from studies on Arabidopsis proteins [6,7,17-23]. Terminal nodes correspond to the following sequences: AtNHX1 - At5g27150; AtNHX2 - At3g05030; AtNHX3 - At3g06370; AtNHX4 - At5g55470; AtNHX5 - At1g54370; AtNHX6 - At1g79610; OsNHX1 - Os07g47100; OsNHX2 - Os05g05590; OsNHX3 - Os11g42790; OsNHX4 - Os06g21360; OsNHX5 - Os09g11450; ScNHX1 - YDR456W.

*NHX* and *SOS1* plant gene families exhibit markedly different evolutionary histories - while the *NHX* family expanded and developed functionally specialized members throughout the history of the land plants (Figures 1 and 2), the *SOS1* family remained a low copy gene family (Figures 3 and 4). It is clear that after gene duplication events, the NHX family members have undergone a series of protein subcellular relocalization and spatial subfunctionalization events. This can be observed by phylogenetically mapping functional and expression information collected from studies on Arabidopsis proteins (Figure 6) [6,7,17-23]. Rice (*Oryza sativa*) proteins also exhibit functional differentiation, although they are not always functionally equivalent to their Arabidopsis homologues. For example, both *AtNHX5* and *OsNHX5*, which are both part of clade 1 (Figure 1), have higher affinity to K^+^ compared to Na^+^[6,40], although AtNHX5 appears to be unresponsive to ABA [22] while OsNHX5 responds to this hormone [40].

In contrast, the *SOS1* genes are found only as single- or low-copy genes in most plant species, suggesting either low duplication rates or a higher rate of gene deletion after gene duplication events for these loci. Although it is unclear why *SOS1* is constrained to be a single- or low-copy gene family, the few cases where a second SOS1-like gene has remained in the genome, it appears to have undergone neofunctionalization. For example, Arabidopsis *SOS1*B is only able to transport Li^+^[16], and the *C*. *nodosa* SOS1B is described as having a different function from CnSOS1A [45]. Moreover, we found a difference in dN/dS between proteins that are diverging in protein length, thus supporting the observed neofunctionalization or possible loss of function of a second copy of *SOS1*. This difference in dN/dS seems to imply that one of the *SOS1* gene copies suffers, more frequently, a non-synonymous mutation resulting in probable loss of function, and more rarely in neofunctionalization.

In our work, we do not detect a relaxation of selective constraint after duplication in the *NHX* gene family, in agreement with a previous study [44]. Furthermore, the difference in average dN/dS between NHX (~0.08) and SOS1 proteins (~0.16) supports previous results [50,52] indicating that in eukaryotes, old duplicated genes evolve slower than singletons, despite an initial relaxation of constraint right after duplication. Our hypothesis is that while NHX proteins can be duplicated and subfunctionalized, they must retain their basic function and thus are under stronger purifying selection. In contrast, SOS1 proteins cannot be as easily subfunctionalized and any duplicate copy seems to be preferentially lost and thus exhibiting more relaxed selective pressures.

Additionally, it may be that the evolutionary constraint on SOS1 proteins arises from fewer possibilities for subfunctionalization, in contrast to cytoplasmatic membrane proteins such as the NHXs. In a simplistic view, this might occur because intracellular membrane proteins can have multiple locations within a cell without detriment to the function it performs, while a plasma membrane protein that is not localized to the plasma membrane would be unable to perform its desired function. Moreover, after subcellular relocalization within the cell, it may be easier to vary transporter affinity towards different ions, and intracellular membrane proteins can then be further subfunctionalized according to their specific location within the cell. This is observed for the NHX family in which AtNHX1-4 have equal affinities towards Na^+^ and K^+^, while AtNHX5 and 6 have a higher affinity towards K^+^ (Figure 6).

### Different means of achieving stress adaptation through *NHX* and *SOS1* duplications

NHX and SOS1 plant protein families have two contrasting evolutionary histories that seem to be related to their protein function and location within the cell. It is especially interesting to assess how the evolution of these two gene families, while different, has resulted in plant adaptation to stress conditions. Our hypothesis is that the first NHX protein was localized in the endosomal compartment of the Golgi and trans-Golgi membranes, corroborating a previous suggestion by Chanroj et al. [39]. The *NHX* duplications, however, allowed the appearance of other NHX proteins that were targeted to the tonoplast. Consequently, an organism with an improved ability to tolerate salt stress arose, since it was able to better retain K^+^ after stress induction [36,37].

On the other hand, the duplication followed by neofunctionalization of SOS1 has generated an increased capacity to tolerate soils with high Li^+^ content in the case of Arabidopsis and related species. This is especially relevant for the Arabidopsis wild relative, Thellungiella spp., that lives in habitats with naturally high Li^+^ concentrations [2]; indeed, *Thellungiella parvula* appears to have three copies of *SOS1B*, probably due to environmental adaptation [2]. Altogether, our results represent two examples of different molecular evolutionary trajectories in land plant genomes that result in organismal stress adaptation through gene duplication.

## Methods

### NHX and SOS1 protein sequences

We used rice protein sequences OsNHX1 (Os07g47100) and OsSOS1A (Os12g44360), in Node Consensus BLASTp of Phytozome v7.0 [53] to obtain the homologous sequences from several plant species (from mosses to eudicots). Sequences belonging to families with an e-value lower than 10^-100^ were used in the analysis. Independent BLASTp searches in Phytozome v7.0 and in NCBI (http://www.ncbi.nlm.nih.gov/) allowed retrieval of homologous sequences of OsNHX1 from the algae *Chlamydomonas reinhardtii* and the gymnosperm *Picea sitchensis*. In these particular cases, sequences with an e-value lower than 10^-10^ were used. Additionally, we also retrieved from NCBI sequences identified as being NHX- or SOS1-like from the yeast *Saccharomyces cerevisiae* (ScNHX1 – YDR456W) and from salt tolerant plant species (in grey background in Additional file 2: Table S1).

In order to improve the quality of the alignments obtained, sequences shorter than 500 or longer than 940 or 1200 amino acids from the NHX and SOS1 families, respectively, were removed from the study (38 out of 158 NHX like sequences, and 6 out of 38 SOS1 like sequences, were excluded). This exclusion was performed because the alignments these sequences produced had a large number of discontinuous aligned stretches and we had low confidence on the homology assignments from these alignments. The identifiers of all the sequences used, and their respective database origin, are listed in the Additional file 2: Table S1.

### Phylogenetic analysis

The phylogeny of NHX proteins was obtained from 121 sequences in 33 different species (Table 1), with the NHX1 protein from the yeast *Saccharomyces cerevisiae* as the outgroup sequence. The SOS1 phylogeny was estimated from 32 sequences in 23 plant species (Table 1), with the sequences from the moss *P*. *patens* used as outgroups. These outgroups were chosen because the *S*. *cerevisiae* sequence (NHA1) most similar to the plant SOS1 genes proved difficult to align with the rest of the plant genes.

Alignments of protein sequences were obtained using MUSCLE [54] and the alignments were refined using Gblocks (default settings) [55,56] in order to minimize the number of positions with missing information (gaps). For each cleaned or non-cleaned alignment the best-fit amino acid substitution model was selected using ProtTest from MEGA5 [57]. Since the Jones-Taylor-Thornton model [58] together with a discrete approximation of the gamma distribution (JTT + Г) was always within the top five best-fit models, we selected this model to obtain the phylogenies using the neighbor-joining (NJ) method [59]. We ran the NJ analysis with 1000 bootstrap replicates in MEGA5. Majority-rule consensus trees of 100 bootstrap replicates were also obtained from maximum likelihood (ML) phylogenies obtained using the JTT model plus a gamma rate distribution approximated using 5 categories. For this we used the SEQBOOT, PROML and CONSENSE programs from PHYLIP 3.69 (http://evolution.genetics.washington.edu/phylip.html). Branches with a good support (>75% bootstrap) were generally the same in the consensus trees obtained using either NJ (data not shown) or ML, although generally ML resulted in higher bootstrap support values.

### Estimation of duplication history

Using the information on species relationships available at the Angiosperm Phylogeny Site (http://www.mobot.org/mobot/research/apweb/) we constructed a cladogram representing the species tree (Additional file 1: Figure S3). Estimation of the gene duplication and loss history of both protein families was performed, using NOTUNG v2.6 [60,61], through reconciliation of the species tree with the NHX and SOS1 ML gene trees (Figures 1 and 2 and Additional file 1: Figure S1 and S2). In order to obtain the most parsimonious estimation of duplication and loss events, the reconciliation of the species and gene trees was followed by rooting, and rearranging branches with less than 75% bootstrap support.

### Determination of selective pressure on amino acid sites

NHX protein sequences were separated into two groups according to the Arabidopsis proteins with which they grouped in the estimated phylogeny. Sequences grouping with Arabidopsis NHX1-4 formed group I, and sequences grouping with Arabidopsis NHX5 and 6 formed group II. Each group was used to obtain a new alignment and a new NJ tree as previously explained. Additionally, for SOS1 sequences three other alignments were obtained in which only orthologs were included or sequences deleted at the N- or C-terminus were excluded. All these alignments were cleaned and analyzed in order to maximize the alignment positions tested. Afterwards, in order to obtain codon-cleaned alignments, we mapped the coding sequences (CDS) of *NHX* and *SOS1* genes to protein alignments.

The codon-cleaned alignments and the NJ phylogenetic trees (both rooted and unrooted), were further used in Codeml from the PAML package [62] to determine heterogeneous selective pressure on amino acid sites. Codon substitution models [41] M0, M1a, M2a, M3, M7, and M8 were applied to the alignments. Each model builds on the preceding one by adding new dN/dS classes. M0 assumes that all sites in an alignment are under purifying selection (dN/dS < 1), M1a allows for some sites to be under neutral selection (dN/dS = 1), and M2a allows for some sites to be under positive selection (dN/dS > 1). M3, M7, and M8 have increased classes of sites that are allowed to be under different selection pressures, being M8 the most complex model with 13 classes of sites. All models were tested at least twice to check for convergence problems and all alignments analyzed had an average synonymous substitution (dS) rate less than 14, which is near the lower limit of the dS range estimated to be disruptive for this type of analyses [63,64]. Afterwards, Likelihood Ratio Tests (LRT) [65] and Akaike Information Criteria (AIC) [66] methods were used to determine the model that best described the data. Neutral or positive selected sites were accepted when model M8 was the best-fit model to the alignment and when sites came up both in model M2 and M8, with a probability ≥ 95% in model M8.

### Comparison of non-synonymous/synonymous rate ratio between clades

Branch-site model [67] from Codeml (PAML) was used to compare the non-synonymous/synonymous substitution rate ratio (dN/dS) between clades or sequences. Bonferroni correction [68] was used to examine significance under these multiple tests. For the SOS1 alignment, the free-ratio model [51] was also used to estimate an independent dN/dS per branch. In both cases, models were tested at least twice to check for convergence problems. Convergence might be an issue, especially for parameter rich models, when there is lack of information in the data, normally resulting from highly similar or divergent sequences (see PAML manual).

## Abbreviations

NHX: Sodium hydrogen exchanger; SOS: Salt overly sensitive; dN: Number of non-synonymous substitutions per non-synonymous site; dS: Number of synonymous substitutions per synonymous site; dN/dS: Non-synonymous/synonymous substitution rate ratio.

## Competing interests

The authors declare that they have no competing interests.

## Authors’ contributions

ISP participated in the design of the study, carried out the computational analysis, and drafted the manuscript. MMP helped carrying out the computational analysis. IAA performed the analysis of the *Arabidopsis thaliana* SOS1A secondary and tertiary structure, looking specifically at the sites predicted to be under neutral or positive selection in the SOS1 proteins. SN, MMO, and MDP conceived the study, participated in its design and coordination, and helped to draft the manuscript. All authors read and approved the final manuscript.

## Supplementary Material

Additional file 1: Figures S1-S5 Figures depicting NHX and SOS1 phylogenies; the species tree used for reconciliation of gene trees; SOS1 phylogenetic tree where branch lengths represent non-synonymous/synonymous rate ratios (dN/dS); and SOS1 phylogenetic tree where branch lengths represent the rate of synonymous substitutions. Click here for file

Additional file 2: Table S1 List of all sequences used in this study with reference to the database from which they were retrieved. Known salt tolerant plant species are highlighted in grey background. A simplified version (without special characters and limited to 10 characters) of the original sequences’ identifiers was used in this study, since the majority of phylogenetic software have limitations for sequence names. Click here for file

## References

[B1] Dassanayake M Oh D-h Hong H Bohnert HJ Cheeseman JM Transcription strength and halophytic lifestyle Trends Plant Sci 2011 16 1 1 3 10.1016/j.tplants.2010.10.006 21094076

[B2] Oh D-H Dassanayake M Bohnert HJ Cheeseman JM Life at the extreme: lessons from the genome Genome Biol 2012 13 241 10.1186/gb4003 22390828 PMC3439964

[B3] Flowers TJ Galal HK Bromham L Evolution of halophytes: multiple origins of salt tolerance in land plants Funct Plant Biol 2010 37 604 612 10.1071/FP09269

[B4] Zhu J-K Plant salt tolerance Trends Plant Sci 2001 6 2 66 71 10.1016/S1360-1385(00)01838-0 11173290

[B5] Apse MP Blumwald E Na^+^ transport in plants FEBS Lett 2007 581 12 2247 2254 10.1016/j.febslet.2007.04.014 17459382

[B6] Venema K Belver A Marín-Manzano MC Rodríguez-Rosales MP Donaire JP A Novel Intracellular K^+^/H^+^ Antiporter Related to Na^+^/H^+^ Antiporters Is Important for K^+^ Ion Homeostasis in Plants J Biol Chem 2003 278 25 22453 22459 10.1074/jbc.M210794200 12695519

[B7] Apse MP Sottosanto JB Blumwald E Vacuolar cation/H^+^ exchange, ion homeostasis, and leaf development are altered in a T-DNA insertional mutant of *AtNHX1*, the *Arabidopsis* vacuolar Na^+^/H^+^ antiporter Plant J 2003 36 229 239 10.1046/j.1365-313X.2003.01871.x 14535887

[B8] Bowers K Levi BP Patel FI Stevens TH The Sodium/Proton Exchanger Nhx1p Is Required for Endosomal Protein Trafficking in the Yeast *Saccharomyces cerevisiae* Mol Biol Cell 2000 11 4277 4294 10.1091/mbc.11.12.4277 11102523 PMC15072

[B9] Brett CL Tukaye DN Mukherjee S Rao R The yeast endosomal Na^+^(K^+^)/H^+^ exchanger Nhx1 regulates cellular pH to control vesicle trafficking Mol Biol Cell 2005 16 3 1396 1405 10.1091/mbc.E04-11-0999 15635088 PMC551501

[B10] Apse MP Salt Tolerance Conferred by Overexpression of a Vacuolar Na^+^/H^+^ Antiport in Arabidopsis Science 1999 285 5431 1256 1258 10.1126/science.285.5431.1256 10455050

[B11] Hernandez A Jiang X Cubero B Nieto PM Bressan RA Hasegawa PM Pardo JM Mutants of the *Arabidopsis thaliana* cation/H^+^ antiporter *AtNHX1* conferring increased salt tolerance in yeast: the endosome/prevacuolar compartment is a target for salt toxicity J Biol Chem 2009 284 21 14276 14285 10.1074/jbc.M806203200 19307188 PMC2682876

[B12] Li T Zhang Y Liu H Wu Y Li W Zhang H Stable expression of *Arabidopsis* vacuolar Na^+^/H^+^ antiporter gene *AtNHX1*, and salt tolerance in transgenic soybean for over six generations Chin Sci Bull 2010 55 12 1127 1134 10.1007/s11434-010-0092-8

[B13] Cao D Hou W Liu W Yao W Wu C Liu X Han T Overexpression of *TaNHX2* enhances salt tolerance of ‘composite’ and whole transgenic soybean plants Plant Cell Tissue Organ Cult 2011 107 3 541 552 10.1007/s11240-011-0005-9

[B14] Brett CL Donowitz M Rao R Evolutionary origins of eukaryotic sodium/proton exchangers Am J Physiol Cell Physiol 2005 288 C223 C239 10.1152/ajpcell.00360.2004 15643048

[B15] Shi H Ishitani M Kim C Zhu JK The *Arabidopsis thaliana* salt tolerance gene *SOS1* encodes a putative Na^+^/H^+^ antiporter PNAS 2000 97 12 6896 6901 10.1073/pnas.120170197 10823923 PMC18772

[B16] An R Chen QJ Chai MF Lu PL Su Z Qin ZX Chen J Wang XC *AtNHX8*, a member of the monovalent cation: proton antiporter-1 family in *Arabidopsis thaliana*, encodes a putative Li/H antiporter Plant J 2007 49 4 718 728 10.1111/j.1365-313X.2006.02990.x 17270011

[B17] Pardo JM Cubero B Leidi EO Quintero FJ Alkali cation exchangers: roles in cellular homeostasis and stress tolerance J Exp Bot 2006 57 5 1181 1199 10.1093/jxb/erj114 16513813

[B18] Bassil E Ohto MA Esumi T Tajima H Zhu Z Cagnac O Belmonte M Peleg Z Yamaguchi T Blumwald E The *Arabidopsis* intracellular Na^+^/H^+^ antiporters NHX5 and NHX6 are endosome associated and necessary for plant growth and development Plant Cell 2011 23 1 224 239 10.1105/tpc.110.079426 21278129 PMC3051250

[B19] Bassil E Tajima H Liang Y-C Ohto M-a Ushijima K Nakano R Esumi T Coku A Belmonte M Blumwald E The Arabidopsis Na^+^/H^+^ Antiporters NHX1 and NHX2 Control Vacuolar pH and K^+^ Homeostasis to Regulate Growth, Flower Development, and Reproduction Plant Cell 2011 23 9 3482 3497 10.1105/tpc.111.089581 21954467 PMC3203450

[B20] Venema K Quintero FJ Pardo JM Donaire JP The arabidopsis Na^+^/H^+^ exchanger AtNHX1 catalyzes low affinity Na^+^ and K^+^ transport in reconstituted liposomes J Biol Chem 2002 277 4 2413 2418 10.1074/jbc.M105043200 11707435

[B21] Aharon GS Apse MP Duan S Hua X Blumwald E Characterization of a family of vacuolar Na^+^/H^+^ antiporters in *Arabidopsis thaliana* Plant Soil 2003 00 245 256

[B22] Yokoi S Quintero FJ Cubero B Ruiz MT Bressan RA Hasegawa PM Pardo JM Differential expression and function of *Arabidopsis thaliana* NHX Na^+^/H^+^ antiporters in the salt stress response Plant J 2002 30 5 529 539 10.1046/j.1365-313X.2002.01309.x 12047628

[B23] Li HT Liu H Gao XS Zhang H Knock-out of *Arabidopsis AtNHX4* gene enhances tolerance to salt stress Biochem Biophys Res Commun 2009 382 3 637 641 10.1016/j.bbrc.2009.03.091 19306843

[B24] Fukuda A Nakamura A Tagiri A Tanaka H Miyao A Hirochika H Tanaka Y Function, Intracellular Localization and the Importance in Salt Tolerance of a Vacuolar Na^+^/H^+^ Antiporter from Rice Plant Cell Physiol 2004 45 2 146 159 10.1093/pcp/pch014 14988485

[B25] Fukuda A Nakamura A Tanaka Y Molecular cloning and expression of the Na^+^/H^+^ exchanger gene in *Oryza sativa* Biochimica et Biophysica Acta - Gene Structure and Expression 1999 1446 1–2 149 155 10.1016/s0167-4781(99)00065-2 10395929

[B26] Li M Lin X Li H Pan X Wu G Overexpression of *AtNHX5* improves tolerance to both salt and water stress in rice (*Oryza sativa* L.) Plant Cell Tissue Organ Cult 2011 107 2 283 293 10.1007/s11240-011-9979-6

[B27] Xue ZY Zhi DY Xue GP Zhang H Zhao YX Xia GM Enhanced salt tolerance of transgenic wheat (*Tritivum aestivum* L.) expressing a vacuolar Na^+^/H^+^ antiporter gene with improved grain yields in saline soils in the field and a reduced level of leaf Na^+^ Plant Sci 2004 167 4 849 859 10.1016/j.plantsci.2004.05.034

[B28] Mahajan S Pandey GK Tuteja N Calcium- and salt-stress signaling in plants: shedding light on SOS pathway Arch Biochem Biophys 2008 471 2 146 158 10.1016/j.abb.2008.01.010 18241665

[B29] Qiu QS Guo Y Dietrich MA Schumaker KS Zhu JK Regulation of SOS1, a plasma membrane Na^+^/H^+^ exchanger in *Arabidopsis thaliana*, by SOS2 and SOS3 PNAS 2002 99 12 8436 8441 10.1073/pnas.122224699 12034882 PMC123085

[B30] Quintero FJ Ohta M Shi H Zhu JK Pardo JM Reconstitution in yeast of the *Arabidopsis* SOS signaling pathway for Na^+^ homeostasis PNAS 2002 99 13 9061 9066 10.1073/pnas.132092099 12070350 PMC124423

[B31] Maser P Thomine S Schroeder JI Ward JM Hirschi K Sze H Talke IN Amtmann A Maathuis FJM Sanders D Phylogenetic relationships within cation transporter families of *Arabidopsis* Plant Physiol 2001 126 1646 1667 10.1104/pp.126.4.1646 11500563 PMC117164

[B32] Guo KM Babourina O Rengel Z Na(+)/H(+) antiporter activity of the *SOS1* gene: lifetime imaging analysis and electrophysiological studies on Arabidopsis seedlings Physiol Plant 2009 137 2 155 165 10.1111/j.1399-3054.2009.01274.x 19758408

[B33] Qiu QS Guo Y Quintero FJ Pardo JM Schumaker KS Zhu JK Regulation of vacuolar Na^+^/H^+^ exchange in *Arabidopsis thaliana* by the salt-overly-sensitive (SOS) pathway J Biol Chem 2004 279 1 207 215 14570921 10.1074/jbc.M307982200

[B34] Gaxiola RA Rao R Sherman A Grisafi P Alper SL Fink GR The *Arabidopsis thaliana* proton transporters, AtNhx1 and Avp1, can function in cation detoxification in yeast Proc Natl Acad Sci USA 1999 96 4 1480 1485 10.1073/pnas.96.4.1480 9990049 PMC15488

[B35] Leidi EO Barragan V Rubio L El-Hamdaoui A Ruiz MT Cubero B Fernandez JA Bressan RA Hasegawa PM Quintero FJ The AtNHX1 exchanger mediates potassium compartmentation in vacuoles of transgenic tomato Plant J 2010 61 3 495 506 10.1111/j.1365-313X.2009.04073.x 19912566

[B36] Barragan V Leidi EO Andres Z Rubio L De Luca A Fernandez JA Cubero B Pardo JM Ion exchangers NHX1 and NHX2 mediate active potassium uptake into vacuoles to regulate cell turgor and stomatal function in Arabidopsis Plant Cell 2012 24 3 1127 1142 10.1105/tpc.111.095273 22438021 PMC3336136

[B37] Jiang X Leidi EO Pardo JM How do vacuolar NHX exchangers function in plant salt tolerance? Plant Signal Behav 2010 5 7 792 795 10.4161/psb.5.7.11767 20495345 PMC3014531

[B38] Hastings PJ Lupski JR Rosenberg SM Ira G Mechanisms of change in gene copy number Nat Rev Genet 2009 10 8 551 564 10.1038/nrg2593 19597530 PMC2864001

[B39] Chanroj S Wang G Venema K Zhang MW Delwiche CF Sze H Conserved and diversified gene families of monovalent cation/H^+^ antiporters from algae to flowering plants Frontiers in Plant Science 2012 3 1 18 22639643 10.3389/fpls.2012.00025 PMC3355601

[B40] Fukuda A Nakamura A Hara N Toki S Tanaka Y Molecular and functional analyses of rice NHX-type Na^+^/H^+^ antiporter genes Planta 2011 233 1 175 188 10.1007/s00425-010-1289-4 20963607

[B41] Yang Z Nielsen R Goldman N Pedersen AMK Codon-substitution models for heterogeneous selection pressure at amino acid sites Genetics 2000 155 431 449 10790415 10.1093/genetics/155.1.431 PMC1461088

[B42] Sato Y Sakaguchi M Topogenic properties of transmembrane segments of *Arabidopsis thaliana* NHX1 reveal a common topology model of the Na^+^/H^+^ exchanger family J Biochem (Tokyo) 2005 138 4 425 431 10.1093/jb/mvi132 16272136

[B43] Brown CJ Takayama S Campen AM Vise P Marshall TW Oldfield CJ Williams CJ Dunker AK Evolutionary rate heterogeneity in proteins with long disordered regions J Mol Evol 2002 55 104 110 10.1007/s00239-001-2309-6 12165847

[B44] Hudson CM Puckett EE Bekaert M Pires JC Conant GC Selection for higher gene copy number after different types of plant gene duplications Genome Biol Evol 2011 3 1369 1380 10.1093/gbe/evr115 22056313 PMC3240960

[B45] Garciadeblas B Haro R Benito B Cloning of two SOS1 transporters from the seagrass *Cymodocea nodosa*. SOS1 transporters from *Cymodocea* and *Arabidopsis* mediate potassium uptake in bacteria Plant Mol Biol 2007 63 4 479 490 10.1007/s11103-006-9102-2 17103013

[B46] Maughan PJ Turner TB Coleman CE Elzinga DB Jellen EN Morales JA Udall JA Fairbanks DJ Bonifacio A Characterization of *Salt Overly Sensitive 1* (*SOS1*) gene homoeologs in quinoa (*Chenopodium quinoa* Willd.) Genome 2009 52 7 647 657 10.1139/G09-041 19767895

[B47] Quintero FJ Martinez-Atienza J Villalta I Jiang X Kim WY Ali Z Fujii H Mendoza I Yun DJ Zhu JK Activation of the plasma membrane Na/H antiporter Salt-Overly-Sensitive 1 (SOS1) by phosphorylation of an auto-inhibitory C-terminal domain PNAS 2011 108 6 2611 2616 10.1073/pnas.1018921108 21262798 PMC3038701

[B48] Smith SA Donoghue MJ Rates of molecular evolution are linked to life history in flowering plants Science 2008 322 5898 86 89 10.1126/science.1163197 18832643

[B49] Duarte JM Wall PK Edger PP Landherr LL Ma H Pires JC Leebens-Mack J dePamphilis CW Identification of shared single copy nuclear genes in Arabidopsis, Populus, Vitis and Oryza and their phylogenetic utility across various taxonomic levels BMC Evol Biol 2010 10 61 10.1186/1471-2148-10-61 20181251 PMC2848037

[B50] Jordan IK Wolf YI Koonin EV Duplicated genes evolve slower than singletons despite the initial rate increase BMC Evol Biol 2004 4 22 10.1186/1471-2148-4-22 15238160 PMC481058

[B51] Geng S Zhao Y Tang L Zhang R Sun M Guo H Kong X Li A Mao L Molecular evolution of two duplicated CDPK genes CPK7 and CPK12 in grass species: a case study in wheat (Triticum aestivum L.) Gene 2011 475 2 94 103 10.1016/j.gene.2010.12.015 21241786

[B52] Davis JC Petrov DA Preferential duplication of conserved proteins in eukaryotic genomes PLoS Biol 2004 2 3 0318 0326 10.1371/journal.pbio.0020055 PMC368158 15024414

[B53] Goodstein DM Shu S Howson R Neupane R Hayes RD Fazo J Mitros T Dirks W Hellsten U Putnam N Phytozome: a comparative platform for green plant genomics Nucleic Acids Res 2012 40 Database issue D1178 1186 22110026 10.1093/nar/gkr944 PMC3245001

[B54] Edgar RC MUSCLE: multiple sequence alignment with high accuracy and high throughput Nucleic Acids Res 2004 32 5 1792 1797 10.1093/nar/gkh340 15034147 PMC390337

[B55] Castresana J Selection of conserved blocks from multiple alignments for their use in phylogenetic analysis Mol Biol Evol 2000 17 540 552 10.1093/oxfordjournals.molbev.a026334 10742046

[B56] Talavera G Castresana J Improvement of phylogenies after removing divergent and ambiguously aligned blocks from protein sequence alignments Syst Biol 2007 56 4 564 577 10.1080/10635150701472164 17654362

[B57] Tamura K Peterson D Peterson N Stecher G Nei M Kumar S MEGA5: molecular evolutionary genetics analysis using maximum likelihood, evolutionary distance, and maximum parsimony methods Mol Biol Evol 2011 28 10 2731 2739 10.1093/molbev/msr121 21546353 PMC3203626

[B58] Jones DT Taylor WR Thornton JM The rapid generation of mutation data matrices from protein sequences CABIOS 1992 8 3 275 282 1633570 10.1093/bioinformatics/8.3.275

[B59] Saitou N Nei M The neighbor-joining method: a new method for reconstructing phylogenetic trees Mol Biol Evol 1987 4 4 406 425 3447015 10.1093/oxfordjournals.molbev.a040454

[B60] Durand D Halldórsson BV Vernot B A hybrid micro–macroevolutionary approach to gene tree reconstruction J Comput Biol 2006 13 2 320 335 10.1089/cmb.2006.13.320 16597243

[B61] Vernot B Stolzer M Goldman A Durand D Reconciliation with non-binary species trees J Comput Biol 2008 15 8 981 1006 10.1089/cmb.2008.0092 18808330 PMC3205801

[B62] Yang Z PAML: a program package for phylogenetic analysis by maximum likelihood CABIOS 1997 13 5 555 556 9367129 10.1093/bioinformatics/13.5.555

[B63] Yang Z On the best evolutionary rate for phylogenetic analysis Syst Biol 1998 47 1 125 133 10.1080/106351598261067 12064232

[B64] Anisimova M Bielawski JP Yang Z Accuracy and power of the likelihood ratio test in detecting adaptive molecular evolution Mol Biol Evol 2001 18 8 1585 1592 10.1093/oxfordjournals.molbev.a003945 11470850

[B65] Yang Z Likelihood ratio tests for detecting positive selection and application to primate lysozyme evolution Mol Biol Evol 1998 15 5 568 573 10.1093/oxfordjournals.molbev.a025957 9580986

[B66] Le SQ Gascuel O An improved general amino acid replacement matrix Mol Biol Evol 2008 25 7 1307 1320 10.1093/molbev/msn067 18367465

[B67] Zhang J Nielsen R Yang Z Evaluation of an improved branch-site likelihood method for detecting positive selection at the molecular level Mol Biol Evol 2005 22 2472 2479 10.1093/molbev/msi237 16107592

[B68] Anisimova M Yang Z Multiple hypothesis testing to detect lineages under positive selection that affects only a few sites Mol Biol Evol 2007 24 5 1219 1228 10.1093/molbev/msm042 17339634

