# Supplementary material for: Different evolutionary histories of two cation/proton exchanger gene families in plants
Source: BMC Plant Biol. 2013 Jul 4;13:97. doi: 10.1186/1471-2229-13-97 (PMC3726471; doi:10.1186/1471-2229-13-97)
Supplement: Additional file 1: Figures S1-S5 — Figures depicting NHX and SOS1 phylogenies; the species tree used for reconciliation of gene trees; SOS1 phylogenetic tree where branch lengths represent non-synonymous/synonymous rate ratios (dN/dS); and SOS1 phylogenetic tree where branch lengths represent the rate of synonymous substitutions. [file 1471-2229-13-97-S1.pdf]

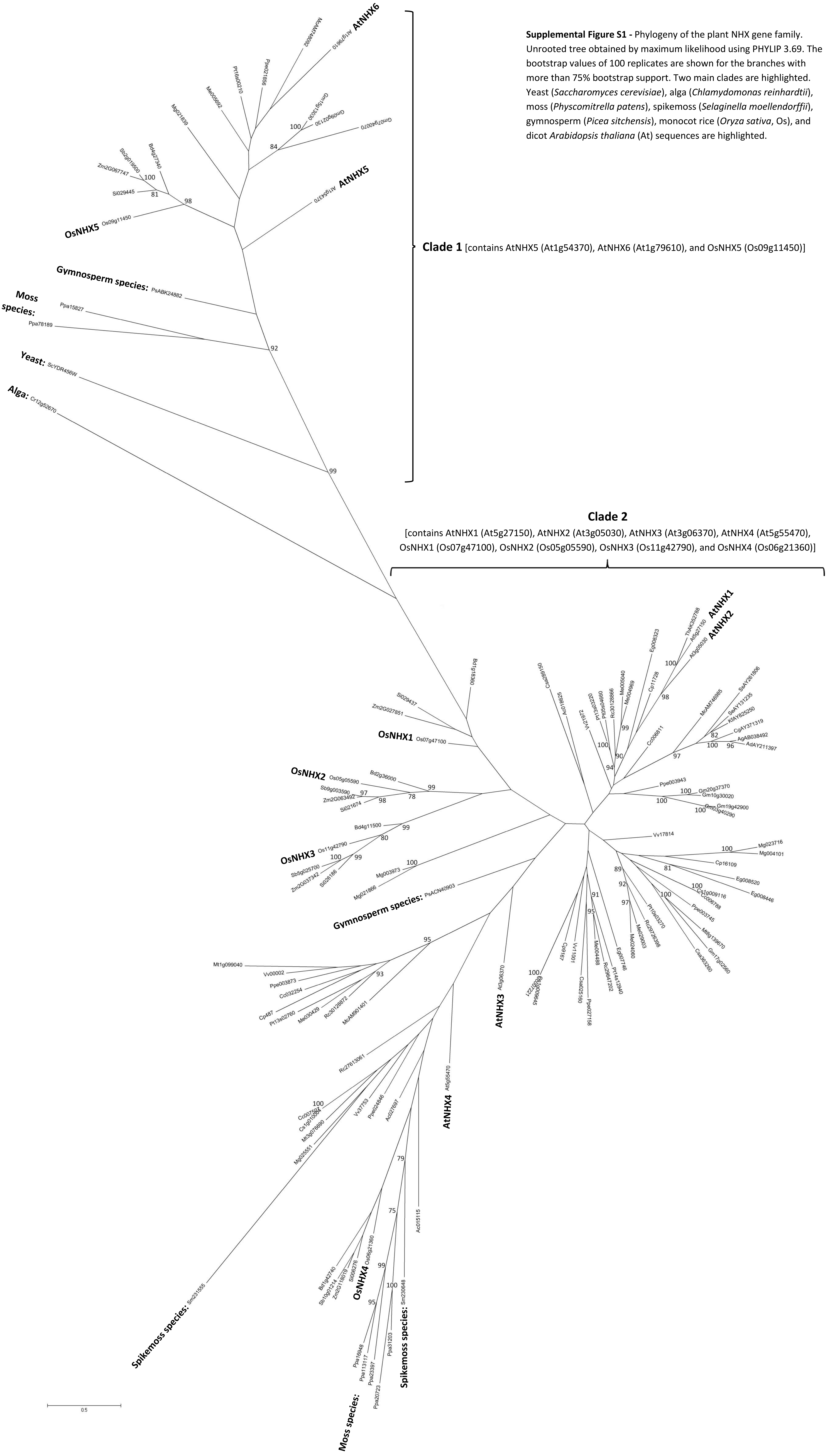

Supplemental Figure S1 - Phylogeny of the plant NHX gene family. Unrooted tree obtained by maximum likelihood using PHYLIP 3.69. The bootstrap values of 100 replicates are shown for the branches with more than 75% bootstrap support. Two main clades are highlighted. Yeast (*Saccharomyces cerevisiae*), alga (*Chlamydomonas reinhardtii*), moss (*Physcomitrella patens*), spikemoss (*Selaginella moellendorffii*), gymnosperm (*Picea sitchensis*), monocot rice (*Oryza sativa*, Os), and dicot *Arabidopsis thaliana* (At) sequences are highlighted.

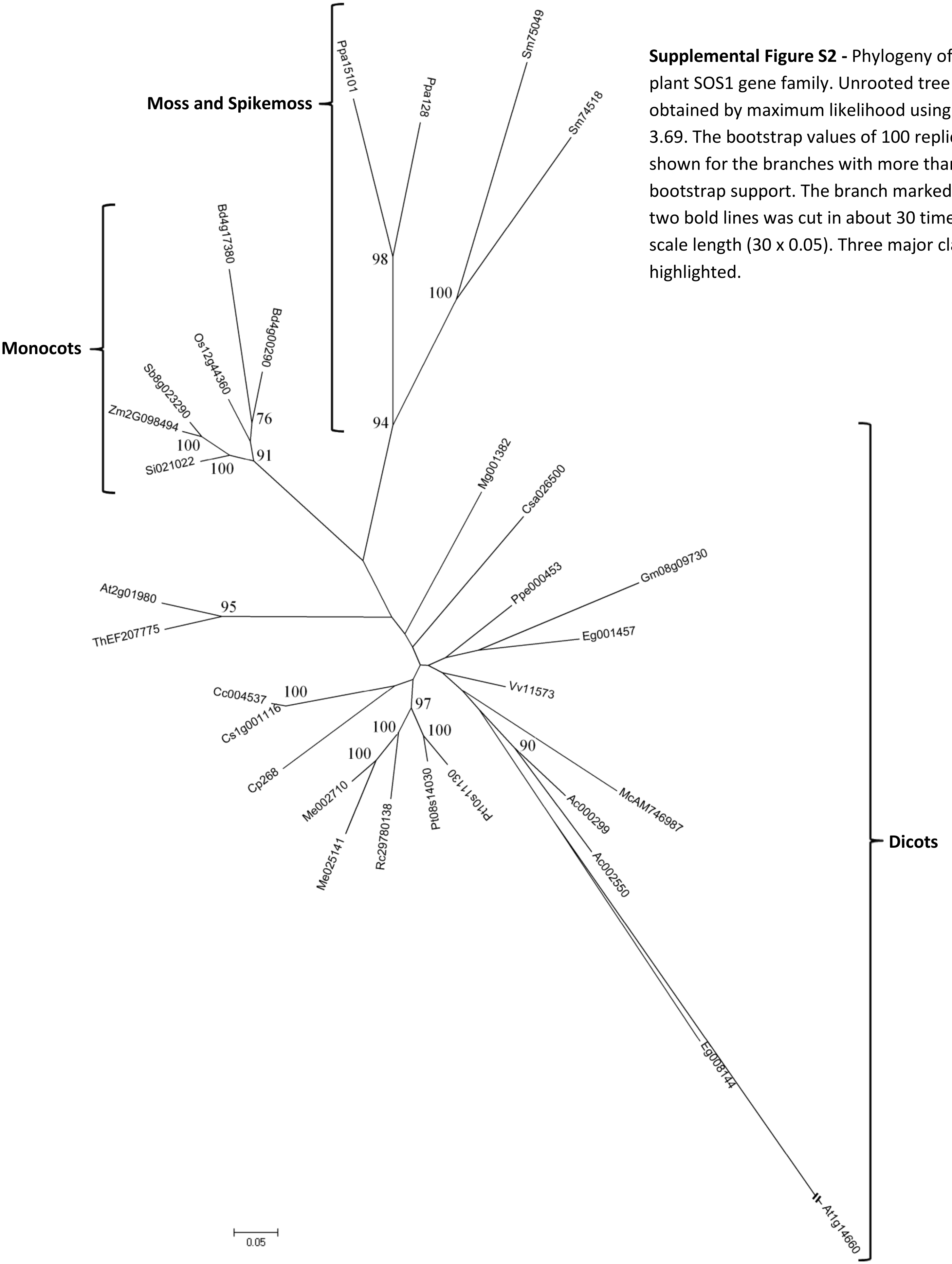

Supplemental Figure S2 - Phylogeny of the plant SOS1 gene family. Unrooted tree obtained by maximum likelihood using PHYLIP 3.69. The bootstrap values of 100 replicates are shown for the branches with more than 75% bootstrap support. The branch marked with two bold lines was cut in about 30 times the scale length (30 x 0.05). Three major clades are highlighted.

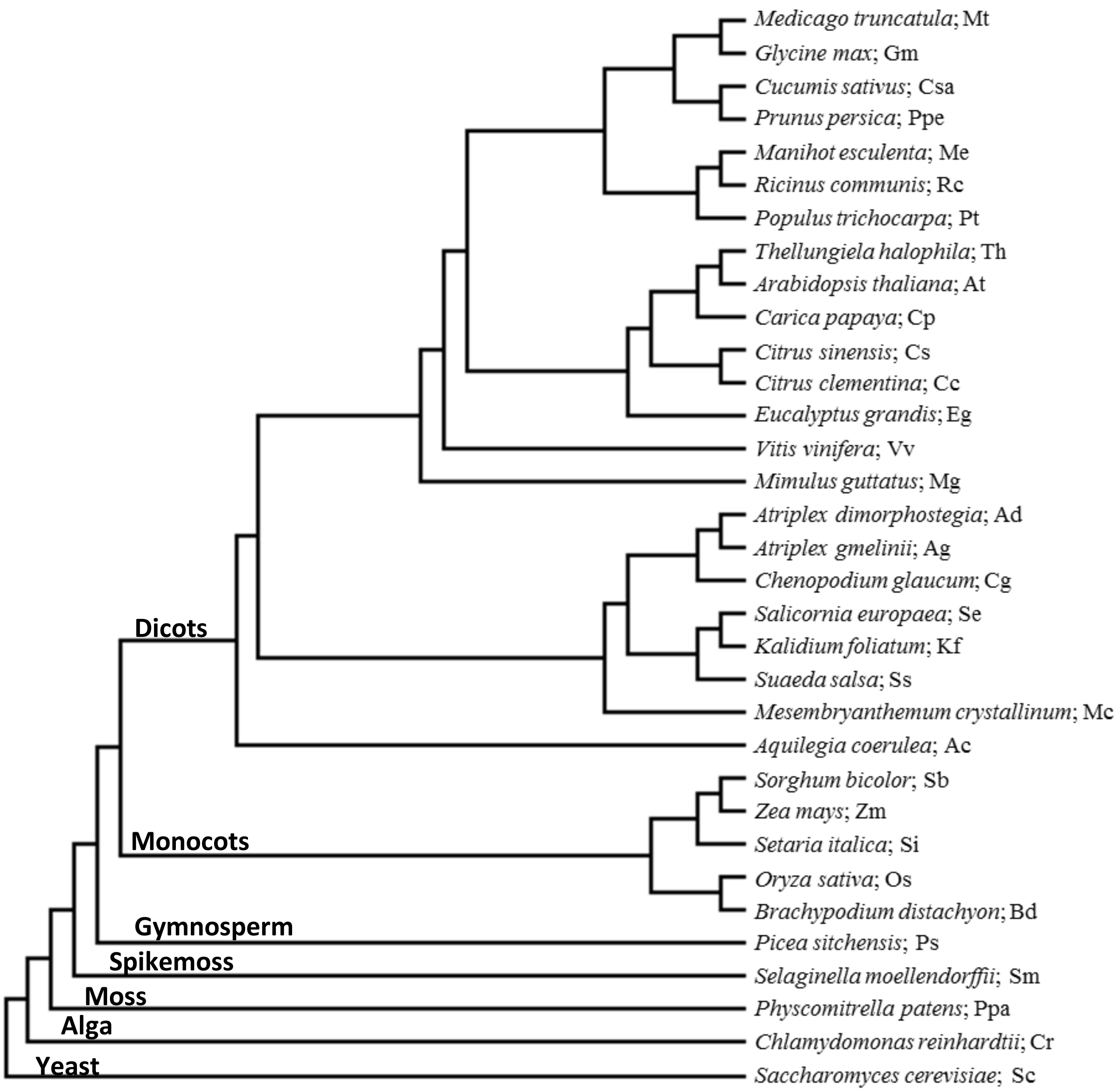

Supplemental Figure S3 - Species phylogeny used for reconciliation with the gene trees. Cladogram generated based on information available at the Angiosperm Phylogeny Site. In front of each species name is the prefix used to identify its sequences.

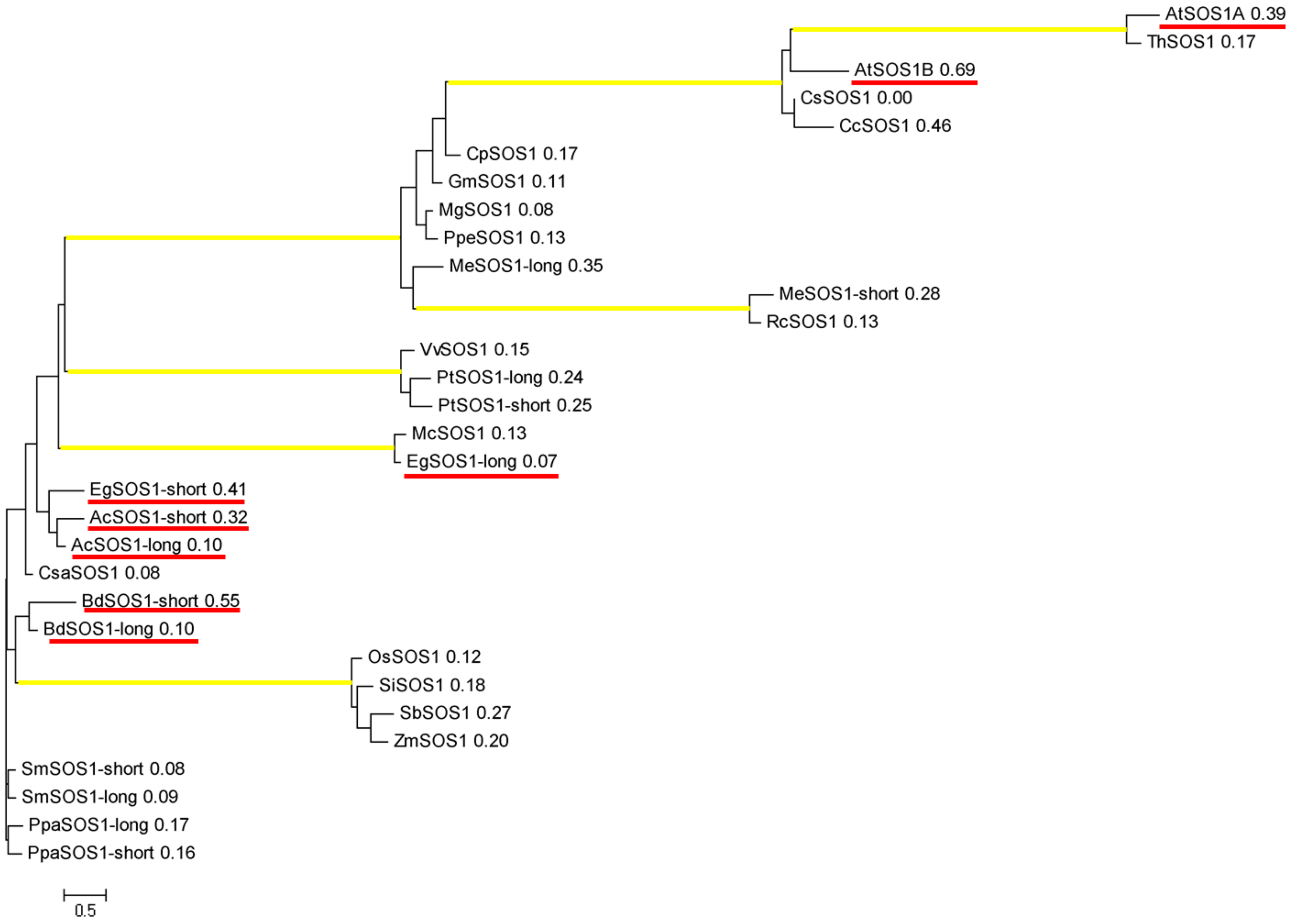

Supplemental Figure S4 - Non-synonymous/synonymous rate ratios SOS1 tree. SOS1 phylogenetic tree where branch lengths represent the non-synonymous/synonymous rate ratios estimated using the free-ratio model in codeml, PAML. The tree was rooted using as outgroup the moss *P. patens* sequences. Underlined external node labels correspond to sequences from species with two putative SOS1-like proteins that differ in protein length in more than 400 amino acid residues and, correspondently, in more than 0.2 of *w* ratio. External nodes are labeled with a prefix dependent on the species (see Additional file 1: Figure S2), with the SOS1 isoform when two exist for the same species (SOS1-long or -short, and A [long] or B [short] for *A. thaliana*), and with the correspondent *w* ratio value. Some internal branches (in yellow) were set to a length (or *w* ratio) of 4, because a very high number was generated by the model which can result from a wrong prediction or from the non-existence of substitutions along those branches.

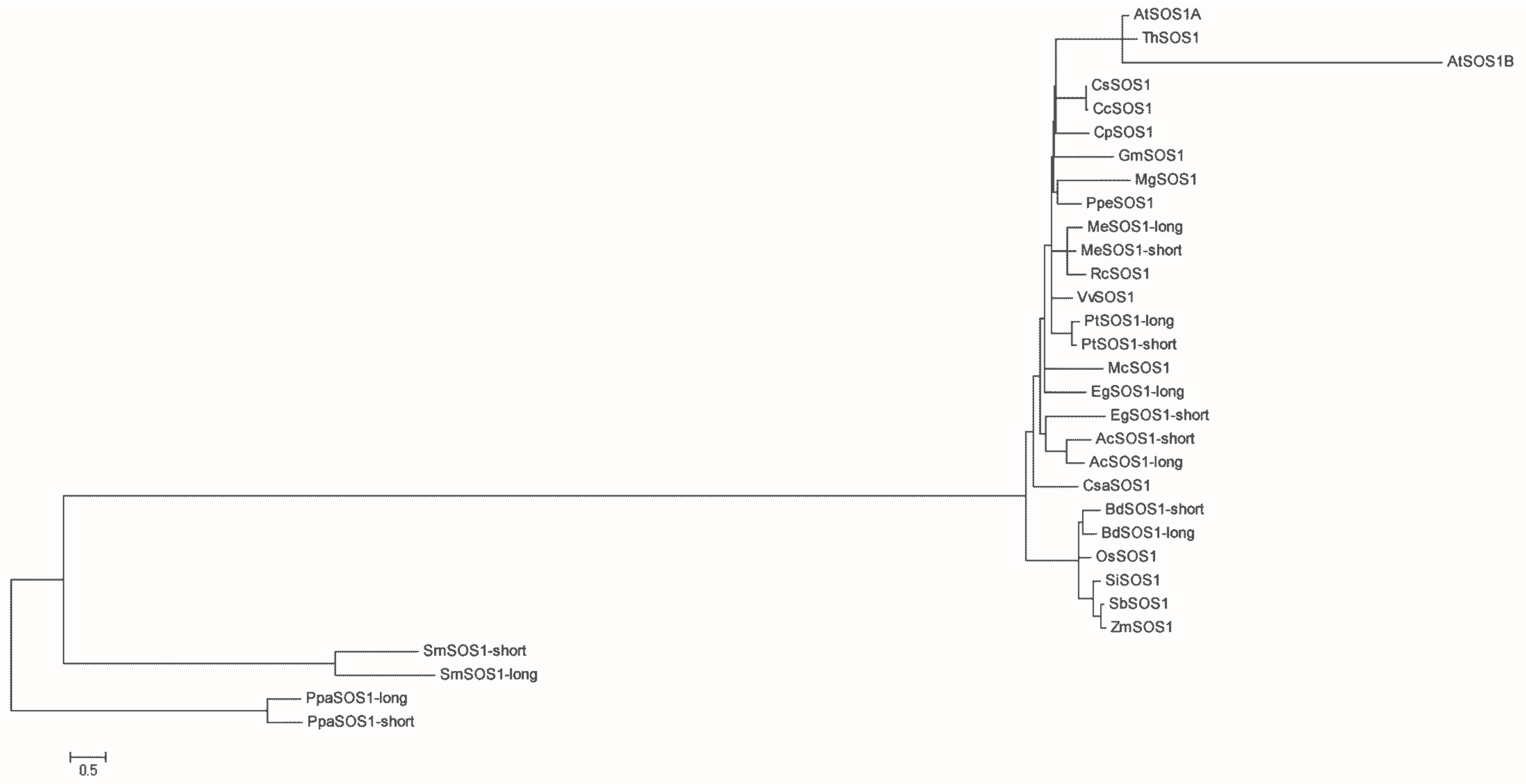

Supplemental Figure S5 - Rate of synonymous substitutions SOS1 tree. SOS1 phylogenetic tree where branch lengths represent the rate of synonymous substitutions estimated using the free-ratio model in codeml, PAML. The tree was rooted using as outgroup the moss *P. patens* sequences. External nodes are labeled with a prefix dependent on the species (see Additional file 1: Figure S2) and with the SOS1 isoform when two exist for the same species (SOS1-long or -short, and A [long] or B [short] for *A. thaliana*).
